# A 36‐Year‐Old Black Male With Sickle Cell Disease and Coexisting Psychiatric Comorbidity: Utilizing Open Dialogue Practices to Overcome Stigma and Enhance Patient‐Centered Care

**DOI:** 10.1155/crps/4014982

**Published:** 2025-12-09

**Authors:** Daniel A. Schaefer, Joseph Stoklosa, Candicee Childs

**Affiliations:** ^1^ Department of Psychiatry, Massachusetts General Hospital, Boston, Massachusetts, USA, harvard.edu; ^2^ Department of Psychiatry, Harvard Medical School, Boston, Massachusetts, USA, harvard.edu; ^3^ Department of Psychiatry, McLean Hospital, Belmont, Massachusetts, USA, mcleanhospital.org

## Abstract

A 36‐year‐old African American male with sickle cell disease (SCD), osteomyelitis, major depressive disorder (MDD), and alcohol use disorder presented with active suicidal ideation following a suicide attempt via increasing alcohol consumption. Diagnostic evaluation suggested MDD. Within the hospitalization, he was also diagnosed with comorbid obsessive‐compulsive disorder. Pharmacologic treatment was initiated with sertraline titrated to 150 mg daily, and his pain was managed with buprenorphine–naloxone 1 mg twice daily, meloxicam 15 mg daily, and acetaminophen 650 mg every 6 h as needed. He also engaged in individual and group psychotherapy, including interventions from psychology and occupational therapy teams, and completed a comprehensive safety plan. Early in the hospitalization, the patient began to share his experiences of mistrust in the healthcare system and stigma as a black patient with SCD. The approach of open dialogue (OD) was then introduced which consisted of providers openly sharing their assessments with the patient present and allowing the patient to provide reflections to further promote transparency and collaboration with patient care. Upon discharge, the patient shared positive feedback on this approach. The patient expressed appreciation for the transparency and collaborative nature of the discussions, which helped to build rapport and reduce mistrust in healthcare institutions. This case illustrates the potential for integrating OD principles to promote transparency and collaborative decision making that could help mitigate the detrimental effects of stigma and structural racism for black patients with SCD. Developing and testing standardized OD protocols could potentially further enhance patient‐centered care and reduce healthcare disparities.

## 1. Introduction

Sickle cell disease (SCD) is the most common monogenic disorder in the United States [1], disproportionately affecting Black Americans [2]. Historically, patients with SCD have encountered persistent stigma and bias in healthcare settings, including assumptions of drug‐seeking behavior and inadequate pain management [3, 4]. Furthermore, psychiatric comorbidities are highly prevalent among individuals with SCD, with depression estimated to affect 28%–36% of patients and anxiety 7%–23% [5–7]. These conditions are often driven by the chronic and unpredictable nature of pain and frequent hospitalizations, which can erode trust between patients and providers. Psychosocial distress in SCD is further associated with poorer adherence, increased healthcare utilization, and diminished quality of life [6].

Standard inpatient psychiatric care for patients with SCD and comorbid psychiatric disorders typically follows a conventional medical model emphasizing symptom stabilization, risk management, and pharmacologic intervention. Family or social networks are incorporated into care planning per patient preference unless there is concern for safety. Care is usually delivered by a multidisciplinary team (psychiatrist, nurse, and social worker) but remains largely clinician‐led, with only intermittent involvement of family or support persons. Psychosocial interventions may be available but are usually secondary to medication management and crisis containment. For patients with SCD, additional attention is paid to pain management and medical comorbidities; however, psychiatric care remains largely standardized to traditional methods of psychiatric care [8].

Open dialogue (OD) is an approach to psychiatric care that emphasizes transparency, shared decision‐making, and the inclusion of the patient’s social network in care discussions. It represents a significant innovation compared to this standard practice. It is a network‐oriented, dialogic approach developed in Finland, which centers on immediate engagement of the patient and their social network (family, friends, peers, or persons deemed supportive by the patient) in joint meetings. All aspects of the clinical situation—including diagnosis, treatment options, and care planning—are discussed openly, with an emphasis on shared decision‐making and transparency. The approach is trauma‐informed, person‐centered, and rights‐based, aiming to empower the patient and their network to direct the narrative and participate actively in recovery [9, 10].

OD has been previously adapted to inpatient psychotic disorders treatment and called “patient‐centered communication” or PCC [11]. No prior reports describe using OD practices counteracting mistrust and enhancing care of patients with SCD. Given OD’s emphasis on transparency, mutual understanding, and collaborative engagement, this approach may be particularly well suited to address the mistrust and systemic inequities that frequently complicate the psychiatric and medical care of patients with SCD.

## 2. Case Presentation

A 36‐year‐old African American male with SCD, osteomyelitis, major depressive disorder (MDD), and alcohol use disorder presented with active suicidal ideation following a suicide attempt via increasing alcohol consumption. His presentation was characterized by markedly depressed mood, pervasive anxiety, poor sleep, diminished energy, and psychomotor retardation. These symptoms developed amid worsening chronic pain from sickle cell crises, recent psychosocial stressors (including a recent interpersonal break‐up), and self‐discontinuation of his prescribed psychiatric medications. Notably, he escalated his alcohol consumption both to numb his pain and with the intent to end his life.

On initial evaluation, the patient was alert and oriented but exhibited a tearful affect. His vital signs were stable, with a blood pressure of 124/76 mmHg, a pulse of 113 beats per min, a temperature of 36.5°C, a respiratory rate of 18 breaths per min, and an oxygen saturation of 95% on room air. Cardiovascular examination revealed a regular rhythm without murmurs, and pulmonary auscultation confirmed clear, bilateral breath sounds. Physical examination was notable for point tenderness over the lumbar spine, consistent with chronic low back pain and history of osteomyelitis. His neurological evaluation was unremarkable. The patient was subsequently voluntarily admitted to the locked inpatient psychiatric unit for further evaluation, medication adjustment and safety.

Diagnostic evaluation suggested MDD precipitated by his suicide attempt, and the Yale–Brown Obsessive‐Compulsive Scale revealed comorbid obsessive‐compulsive disorder. The patient had previously been prescribed sertraline, which was reinitiated and titrated to 150 mg daily by time of discharge without adverse effects, with the anticipation that further dosage optimization may be beneficial given its efficacy in treating OCD. His pain was managed with buprenorphine–naloxone 4 mg/1 mg twice daily, meloxicam 15 mg daily, and acetaminophen 650 mg every 6 h as needed. He also engaged in individual and group psychotherapy, including interventions from psychology and occupational therapy teams, and completed a comprehensive safety plan.

In response to his openness about feeling stigmatized and concerns about how he was being perceived, the treatment team adapted an OD approach. Daily OD network meetings were conducted by a multiprofessional team (psychiatrists, nurse, and social worker), with additional nurse practitioners from the outpatient sickle cell team joining at the patient’s request at subsequent meetings. Each meeting lasted 45–60 min: the first 20–30 min covered introductions, symptom assessment, treatment tolerance, and patient concerns. The dialogic phase followed, in which team members reflected on the patient’s situation in the third person point of view while the patient listened, then the patient shared reflections (~10 min), and the session concluded with collaborative treatment planning. Family and peer involvement were offered per OD protocol but declined by the patient. Adaptations (e.g., no home visits and limited network involvement) reflected patient preference and inpatient context. On discharge following his psychiatric stabilization, the patient expressed positive feedback on the OD process. He admitted in reflection that he had developed a deep mistrust in the medical system, noting the transparency and collaborative nature of the discussions had significantly reduced his anxiety about his treatment and helped him understand himself and his treatment better.

## 3. Discussion

We present a case demonstrating the application of OD practices in managing the challenges of SCD with psychiatric comorbidities. This resulted in increased transparency, better rapport, and less self‐reported mistrust in the medical system.

SCD is frequently complicated by comorbidities such as MDD, anxiety, and chronic pain [8]. For Black patients, these challenges are compounded by structural racism and implicit bias that manifest in stigmatizing language, such as labeling patients “drug‐seeking,” and delayed treatment, such as extended wait times for pain management [3]. This not only compromises the quality of care but also reinforces self‐stigma and erodes trust in healthcare institutions [4].

In our case, the treatment team integrated OD principles into routine rounds without additional formal training, facilitated by one member’s experience with OD. By including the patient in real‐time, transparent discussion and using person‐centered language, the team created a respectful, collaborative space for communication, reducing anxiety, and mistrust engendered by past negative experiences.

OD is defined as “not as a method or technique but as a process of interaction which can be applied to different conditions and circumstances” [12]. It emphasizes transparent communication, immediate engagement, and shared decision making by flattening traditional power structures and empowering patients to become equal partners in their care. The approach promotes personal recovery, communication, and service engagement by ensuring that treatment decisions occur with the individual and their support network, rather than about them. It is guided by seven principles: immediate help, utilizing a social network perspective, flexibility and mobility, responsibility, psychological continuity, tolerance of uncertainty, and dialogue [13]. In practice, these principles were enacted through network meetings or what we consider inpatient rounds, during which the patient, their social network, and the clinician team engage in open, collaborative discussions.

While early evidence supports the benefits of OD in promoting collaborative decision‐making and reducing coercion [14], further randomized controlled trials is needed to standardize these practices [15] and fully evaluate their effect on reducing stigma and systemic inequities [11]. Although OD has been most extensively studied in the context of acute psychiatric crises, particularly psychosis, high‐quality data supporting its use in other diagnostic or medical contexts remain limited. However, this case suggests that OD principles may also hold promise for patients who experience mistrust of the medical system or have had prior negative experiences with traditional models of care, including those with chronic pain or substance use disorders. Integrating OD‐informed approaches into broader consultation‐liaison psychiatry and inpatient medical settings may, thus, hold promise for improving patient engagement and rebuilding therapeutic trust across diverse contexts.

This report describes a single case, and therefore, cannot be generalized to all patients with SCD or psychiatric comorbidity. Quantitative symptom scales (e.g., PHQ‐9, GAD‐7, or standardized pain measures) were not formally collected during this admission, which limits the ability to objectively track changes in mood or pain severity; future applications of OD in similar cases could benefit from integrating such measures to improve reproducibility. In addition, the clinical team had prior familiarity with OD principles, which may limit reproducibility in other settings.

## 4. Conclusion

This case underscores the profound challenges Black patients with SCD face amid psychiatric comorbidities and systemic bias. Integrating OD principles not only improved clinical outcomes but also played a pivotal role in rebuilding trust and transforming his approach to managing chronic pain. By promoting transparency and collaborative decision‐making, OD shows promise as an adjunct to conventional treatment strategies, potentially mitigating the detrimental effects of stigma and structural racism. Future research should focus on developing and testing standardized OD protocols to further enhance patient‐centered care and reduce healthcare disparities.

## Ethics Statement

According to Mass General Brigham institutional policy, single case reports do not require IRB review.

## Consent

Consent was obtained for publication. Patient anonymity has been maintained, and no personally identifiable information is included.

## Disclosure

All the authors provided final approval of the manuscript and agree to be accountable for all aspects of the work.

## Conflicts of Interest

The authors declare no conflicts of interest.

## Author Contributions

All the authors were involved in revising the manuscript critically for important intellectual content.

## Funding

No external funding was received.

## Data Availability

All the data supporting the findings of this study are contained within the manuscript.
